# Information needs for the rapid response team electronic clinical tool

**DOI:** 10.1186/s12911-017-0540-3

**Published:** 2017-10-02

**Authors:** Amelia Barwise, Sean Caples, Jeffrey Jensen, Brian Pickering, Vitaly Herasevich

**Affiliations:** 1 Division of Pulmonary and Critical Care Medicine, Mayo Clinic, Rochester, MN USA; 2 Department of Anesthesiology, Mayo Clinic, Rochester, MN USA

**Keywords:** Rapid response team (RRT), Electronic medical record (EMR), Information overload, Health information technology

## Abstract

**Background:**

Information overload in healthcare is dangerous. It can lead to critical errors and delays. During Rapid Response Team (RRT) activations providers must make decisions quickly to rescue patients from physiological deterioration. In order to understand the clinical data required and how best to present that information in electronic systems we aimed to better assess the data needs of providers on the RRT when they respond to an event.

**Methods:**

A web based survey to evaluate clinical data requirements was created and distributed to all RRT providers at our institution. Participants were asked to rate the importance of each data item in guiding clinical decisions during a RRT event response.

**Results:**

There were 96 surveys completed (24.5% response rate) with fairly even distribution throughout all clinical roles on the RRT. Physiological data including heart rate, respiratory rate, and blood pressure were ranked by more than 80% of responders as being critical information. Resuscitation status was also considered critically useful by more than 85% of providers.

**Conclusion:**

There is a limited dataset that is considered important during an RRT. The data is widely available in EMR. The findings from this study could be used to improve user-centered EMR interfaces.

## Background

Rapid Response teams (RRT) have been critically studied to date, particularly in terms of whether they are indeed effective or not [1, 2]. Delayed RRT activations increase mortality and morbidity outcomes. As well as activating the RRT system effectively and quickly we must also maximize the management strategies the RRT can offer. For those who are not the primary care team during the hospital stay, the task of addressing acute needs without data access and detailed data review, places those responding to an RRT at a great disadvantage. The problems with information overload for providers, has been well documented. The potential concerns from this information overload include [3] the inability of practitioners to discern pertinent [4] from information that is irrelevant and the accumulation of errors of cognition and performance associated with data corruption [5, 6]. Many of the current EMRs may be a barrier to providing ideal patient care. EMRs are often poorly designed for providers, particularly those trying to make rapid assessments and difficult decisions about critically ill patients in times of acute physiological change. A potential approach to alleviate the risk of information overload may hinge on the development and implementation of advanced health information technology (HITs). Both the Institute of Medicine and the United States Department of Health and Human Services have advocated for the enhanced creation and use of efficient EMRs [7]. Interfaces are evolving to try to meet this unmet need and HITECH (HIT for Economic and Clinical Health) Act was established to promote this endeavor through federal grants [8]. To best design those interfaces we require information from providers about what data they find essential during times of acute deterioration. Those at risk or suffering from acute physiological deterioration are a unique group with specific but not necessarily complex medical information needs.

Novel EMR interfaces can be difficult to implement, even in academic medical centers. Usually outcomes improve with fewer errors and increased productivity but there remain concerns about the best way to design and integrate EMRs into clinical practice [5, 9–11].

We conducted a survey among Mayo providers to identify what information they found useful in decision making when they were called to the bedside during RRT activation. The results of this study can shape the way we respond to patients’ physiological needs. Information overload can lead to mistakes and delays, therefore prioritizing essential data that providers want is helpful.

Objective: The primary objective of this study was to assess the base information needs of clinicians responding to a RRT call. How these elements were integrated into the workflow was not addressed nor an objective of this study. From literature reviews we believe this is the first study that attests to elucidate the exact clinical information needs of RRT providers and allied health professionals on the team. In the present study we address this gap in knowledge by using a modified Delphi process utilizing a survey to determine what specific data the Rapid Response team finds useful in guiding clinical decision making for inclusion into future EMR viewer platforms.

## Methods

### Study design

An anonymous web based survey was conducted at Mayo Clinic Rochester, MN, an academic tertiary center where a comprehensive EMR is deployed [12]. The Mayo Clinic has just over 2000 beds in the Rochester location with 135,000 hospital admissions annually. The survey was conducted among RRT providers and ICU providers of varying clinical roles. The Institutional Review Board (IRB) deemed the study a quality improvement project and therefore exempt from IRB approval and not requiring consent.

### Study subjects

The survey was distributed to all potential RRT providers among 4 distinct clinical role designations: attending physicians (AP), physicians-in-training (PT), respiratory therapists (RT) and registered nurses (RN) as well as among anesthesiology residents and consultants who may occasionally attend RRT calls, but are not generally part of the usual RRT structure. The survey was distributed to a total of 395 possible survey participants. We had a total of 97 respondents including 16 attending-consultants, 15 clinical fellows, 21 residents, 27 nurses (RN) and 17 Respiratory Therapists (RT). The consultants were all board certified intensivists, the fellows were all critical and pulmonary care fellows carrying a designated RRT pager, the nurses were all trained and employed in ICU as were the respiratory therapists. All providers were responding to RRT calls during their ICU shifts and their responsibilities also included care of the ICU patients.

### Data collection instruments

We used previously reported clinical data priority information identified from similar studies [5, 13], and compiled a list of 45 available candidate data points considered important for clinical decision making. Using a 5 point Likert scale ranging from *not useful* [1] to *very useful* [5], subjects were asked to rate each of the 45 data items according to importance in helping guide decisions at RRT calls. All data items are included in the current EMR and would have been familiar to the participants. Respondents were also given the opportunity to highlight missing data points with a free-text box included for additional comments.

Items were separated into several different categories for survey display: demographics (8 items), past medical history (6 items), investigations (7 items), laboratory (8 items), infection (8 items), physiology (5 items), other (5 items), allergy (3 items).

### Study procedures

We created the survey using the LimeSurvey *(* *www.limesurvey.org)* *)* web-based stand-alone tool installed inside the Mayo Clinic firewall. We distributed the link to the study participants via email. Email reminders were generated to enhance survey participation. Participants included those potentially involved in the multidisciplinary RRT team (Intensivists, critical care fellows, respiratory therapists and registered ICU nurses, and ICU providers including anesthesiology residents and consultants). The identity of the participants and survey responses were kept confidential, in keeping with Delphi methodology.

#### Data analysis

Survey responses were collected and formulated into tables in MS Excel. We calculated the mean score (MS) for each data item, then ranked the mean score results in descending order to generate median values and interquartile ranges. We also determined the percentage of participants ranking each individual item as [1] *not useful* or *very useful* [5]*.*Each data item mean score was also analyzed and stratified individually to assess if there was any inter-role rating variability. Cumulative means differed across groups with consultants scoring data at the lowest mean overall of 3.8 and “others” giving mean scores to data of 4.7. Fellows, residents, RNs and Respiratory therapists gave mean data scores of 3.9 and 4.0.

#### Statistics

All descriptive statistics and data analysis including comparing means with the Kruskall Wallis test were done using JMP (v 9.0.1, SAS Institute, Cary, NC): *p* < 0.05 was considered statistically significant. To optimize data visualization tableau software (Seattle, WA) was used. Study format, design and statistical analysis were conducted in accordance with similar published studies performed at our institution and under the guidance of statistical support [13, 14].

## Results

Three hundred ninety five survey requests email were emailed with a total of 97 responses obtained, generating a response rate of 24.5%. These included 16 consultants, 15 fellows, 21 residents, 27 nurses (RN) and 17 Respiratory Therapists (RT).

**Fig. 1:**
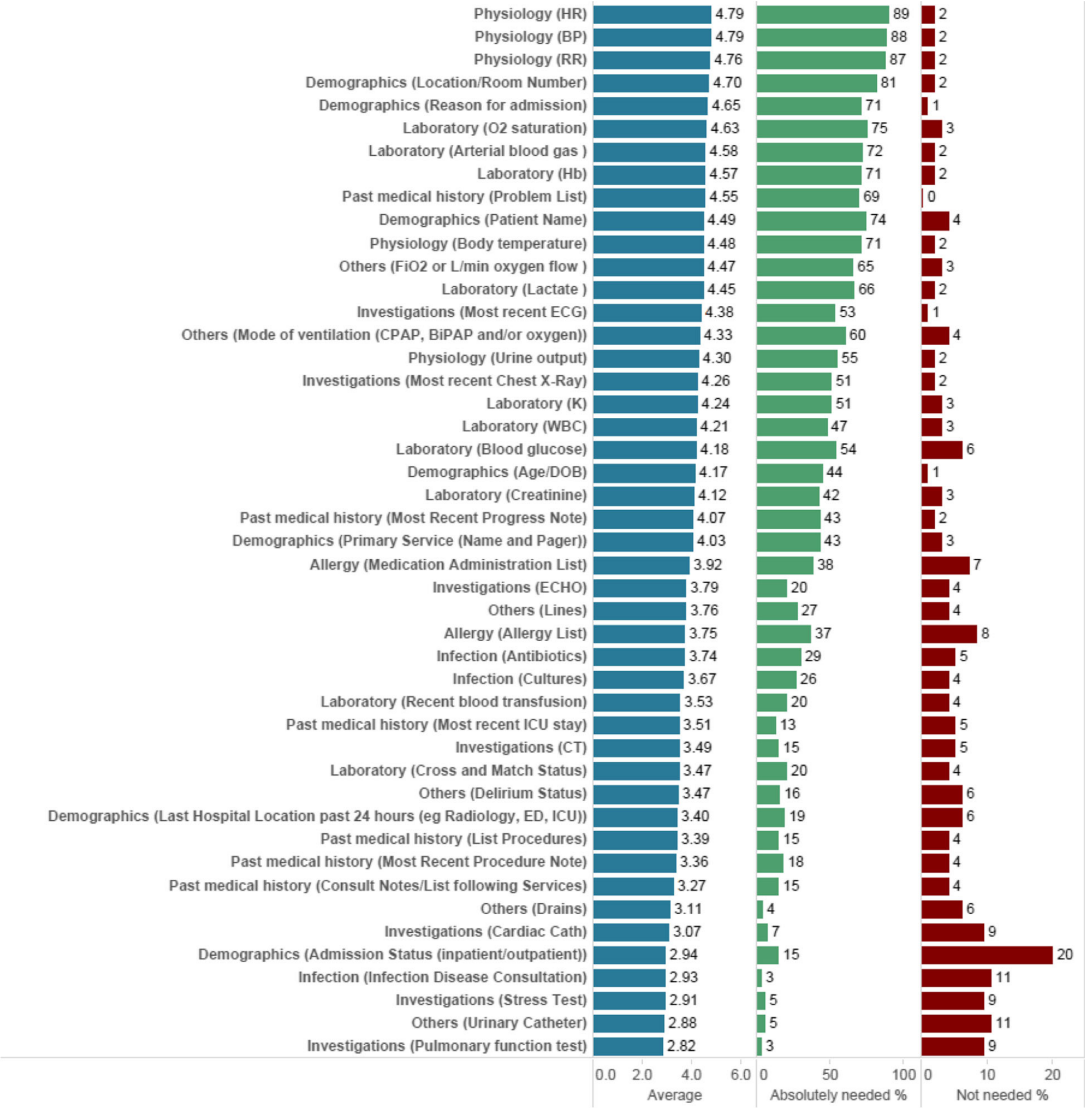
Demonstrates the results of the distribution of mean scores for every data item stratified

Figure 1 shows each of the 47 data items listed by descending mean scores (MS). The 5 items with the highest MS were: *resuscitation status, heart rate, blood pressure, respiratory rate, and location.* The median MS was 4.07 (max 5). Just over 50% of the data items fell within the top 2 quartiles when distributed by proportional quarters.

The lowest 5 rated data items were: *admission status, (inpatient/outpatient), infectious disease consultation, stress test, urinary catheter and pulmonary function tests. (ie. results of these procedures).*

Another element demonstrated in Fig. 1 is the percentage of respondents that rated each item “*not useful” *or “*very useful”.* Resuscitation status received the highest percentage of respondents (91%) rating it as a “*very useful”* data item. Twenty of the 47 items (43%) received a *“very useful” rating by more than 50% of respondents.* The six items with the lowest mean score had the highest percentage of respondents rating each data item as *“not useful” (9–20%)*. Inpatient/outpatient status was rated as “not useful” by 20% of respondents.

Following stratification of individual data item mean scores by clinical roles: consultant, fellow, resident, RN and RT resulted in a change in the order of highest 10 ranking data items and some differences that may reflect the role within the RRT team (example RT focused on ventilator/FiO2 settings) (Table 1). However these top 10 data items included heart rate, blood pressure, respiratory rate, and resuscitation status in all 5 groups. Similarly 3 data items *Pulmonary Function Tests (PFTS), Infectious Disease Consultation, and Urinary Catheter* were among the 10 lowest rated items in all groups.

**Table 1:** Top ten data elements stratified by clinical role

	Consultant	Fellow	Resident	RN	RT
1	HR	BP	HR	Code status	Code status
2	BP	HR	BP	Reason for admission	Rm number/location
3	Problem list	Code status	Code status	Rm number/location	O2
4	RR	Reason for admission	RR	ABG	HR
5	Code status	Problem list	Rm number/location	Hb	RR
6	Hb	RR	Reason for admission	Lactate	BP
7	O2	Hb	Problem list	HR	Others FiO2
8	Name	Name	Name	RR	Others Vent Mode
9	Rm number/location	O2	ABG	O2	ABG
10	Lactate	Lactate	ECG	BP	Chest xray

**Fig. 2:**
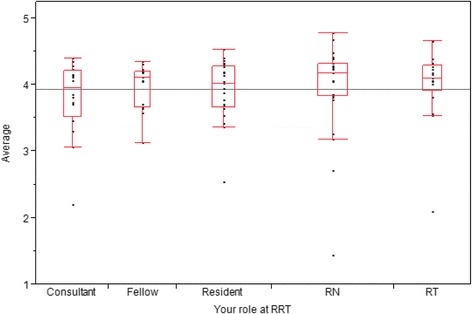
Variance of average score by clinical role. Variance in the individual data item mean scores stratified by clinical role. Data are displayed in box-whisker graphs (median, interquartile range, total range with the horizontal line across the box showing the grand mean)

Figure 2 shows the distribution of mean scores for every data item stratified by clinical roles. The Likert scores for each data element were used to calculate the median and interquartile range of all scores for each respondent category. No statistically significant differences were noted between the various clinical roles when comparison was made using a Wilcoxon test. When analysis of free-text items was performed this did not reveal any additional information not described above.

## Discussion

We conducted a survey among dedicated RRT clinicians and various other ICU clinicians to better understand their clinical information needs when responding to an RRT and to inform future efforts to create a more effective EMR interface for those responding to RRT calls. To our knowledge this is the first study that attempts to assess these specific needs in a systematic format. Our survey had a moderate response rate of just under 25%, with participants distributed among 5 distinct clinical roles-Consultants, Fellows, Residents, Respiratory Therapists and Registered Nurses. However, the low response rate mostly reflects the fact that the distribution list included clinicians who do not frequently participate in RRTs. The data indicates that when making clinical decisions, RRT providers rely on some basic data provided through the EMR as more than half of the data items were ranked within the top 2 proportional quartiles. In addition to this finding, a large difference in the extremes of the rankings was noted, with many more respondents ranking items as absolutely necessary as compared to not needed. The results reflect what one might expect clinically with basic physiological information (heart rate, blood pressure and respiratory rate) as well as resuscitation status being ranked as the most important data items.

RRT clinicians need to be able to access critical information quickly and this information must be pertinent and current. Knowing the patient’s resuscitation status is vital as well as basic physiological parameters including heart rate, blood pressure and respiratory rate. This aligns with what one might expect clinically and underpins the idea that presenting simple but important data will aid the Rapid Response Teams assessment of and ability to rescue patients.

When comparing across different roles on the RRT it is notable that the overall 4 highest ranked items are among the top 10/47 in all roles with some minor differences within the 10 that may reflect specific tasks assumed by those roles (example the RT ranked Vent mode and Fi02 among the top 10 data items where as these were not considered important within the other roles on the team). For the 10 lowest ranked items there was agreement among all groups surveyed that PFTs, presence of urinary catheter and infectious disease consultation were not useful.

The information needs of primary care physicians and the EMR has been studied as attempts are made to conceptualize the ideal user interface for use in consultations in the outpatient setting. A recent study done in a primary care setting which assessed the EHR needs of family medicine and internal medicine physicians in primary care [15] used semistructured interviews to identify the note sections that were relevant to the providers’ needs. This approach differs from what was done in the survey-based study conducted here. Time pressures are also a concern in primary care but the critical and acute nature of the illness seen at RRT activations compared to the normal presentations in primary care make the two studies difficult to compare. Survey research done in the OR revealed the 5 data items with the highest mean score were name, planned procedure, medical record number,SpO2 and NIBP. The same group also studied the information needs of those working in the PACU and found name, medical record number, age, allergies and planned procedure to be the most highly rated items in terms of importance [13]. For those who work in the NICU the top 5 data items with the highest mean score were daily weight, pH, PC02, Fi02 and blood culture results. The needs of the neonatal provider while working daily in a neonatal intensive care unit are unique but the importance of physiological data remains paramount. Our findings are limited as the study was conducted in a single center and the results may not apply in other settings. However with the widespread use of RRTs even in diverse hospital situations it is likely similar data would be helpful to providers when seeing acutely deteriorating patients. The study captured responses from a wide spectrum of clinicians including different clinical roles and level of training so even in other hospital settings which may have different RRT structures it is likely that our results reflect parameters that are important for all potential members of the RRT.

Designing interfaces that can assist in clinical decision making by presenting data that is relevant saves much needed time when patients are acutely physiologically unstable. Ultimately this should improve response effectiveness in Rapid Response Team activations and contribute to improved outcomes for patients. Using this methodological process to assess how we design the new technology so it is tailored to the needs of the users will enhance the likelihood of implementation success [15]. These methodologies can be repeated in other settings to evaluate the information needs of providers. Further research is needed to understand the needs of the RRT including using qualitative methodology and increasing our knowledge of providers’ needs, will be vital for effective interface design and implementation.

## Conclusion

When responding to a RRT event clinical data such as heart rate, respiratory rate, and blood pressure as well as Resuscitation status are ranked as critically useful by more an 85% of providers. This consensus is reassuring and contributes to current knowledge about how we can improve the EMR interface. The results demonstrate evidence that simple real time physiological data is very useful in acute deterioration.

## Abbreviations

EHR: electronic health record
EMR: Electronic Medical Record
HIT: Health Information Technology
ICU: Intensive Care Unit
JMP: Joint Mission Planning
MS: mean score
NICU: neonatal intensive care unit
OR: operating room
PACU: post anesthesia care unit
PFTS: pulmonary function tests
RN: registered nurse
RRT: Rapid Response Team
RT: respiratory therapist
